# Ediacaran Doushantuo-type biota discovered in Laurentia

**DOI:** 10.1038/s42003-020-01381-7

**Published:** 2020-11-06

**Authors:** Sebastian Willman, John S. Peel, Jon R. Ineson, Niels H. Schovsbo, Elias J. Rugen, Robert Frei

**Affiliations:** 1 Department of Earth Sciences (Palaeobiology), Uppsala University, Villavägen 16, SE-752 36 Uppsala, Sweden; 2 Geological Survey of Denmark and Greenland, Øster Voldgade 10, DK-1350 Copenhagen K, Denmark; 3 Department of Geosciences and Natural Resources Management, University of Copenhagen, Øster Voldgade 10, DK-1350 Copenhagen K, Denmark

**Keywords:** Palaeontology, Palaeoecology

## Abstract

The Ediacaran period (635–541 Ma) was a time of major environmental change, accompanied by a transition from a microbial world to the animal world we know today. Multicellular, macroscopic organisms preserved as casts and molds in Ediacaran siliciclastic rocks are preserved worldwide and provide snapshots of early organismal, including animal, evolution. Remarkable evolutionary advances are also witnessed by diverse cellular and subcellular phosphatized microfossils described from the Doushantuo Formation in China, the only source showing a diversified assemblage of microfossils. Here, we greatly extend the known distribution of this Doushantuo-type biota in reporting an Ediacaran *Lagerstätte* from Laurentia (Portfjeld Formation, North Greenland), with phosphatized animal-like eggs, embryos, acritarchs, and cyanobacteria, the age of which is constrained by the Shuram–Wonoka anomaly (c. 570–560 Ma). The discovery of these Ediacaran phosphatized microfossils from outside East Asia extends the distribution of the remarkable biota to a second palaeocontinent in the other hemisphere of the Ediacaran world, considerably expanding our understanding of the temporal and environmental distribution of organisms immediately prior to the Cambrian explosion.

## Abstract (web summary)

The Doushantuo biota of China is incredibly important for understanding the evolution of complex animals. Willman et al. report on evidence of Doushantuo-like biota from the Ediacaran of North Greenland, indicating that these important organisms were distributed across multiple palaeocontinents.

## Introduction

Remarkably detailed preservation of cells and soft tissues has been described from several Precambrian *Lagerstätten*, providing some of the best documented examples of early organismal evolution^1–3^. Typically, this quality of preservation is made possible through several pathways, of which diagenetic phosphate replacement of originally organic material (“Doushantuo-type preservation”) has provided some of the most spectacular descriptions of putative animal embryos, acritarchs, and small shelly fossils across the Ediacaran–Cambrian boundary^4–6^. Following their discovery and rise to fame in the late 1990s, the fossils from the well-known Ediacaran Doushantuo Formation of China have sparked controversy and debate, especially regarding speculative interpretation as fossil animal embryos^5,7–13^. However, in contrast to the widespread localities yielding macroscopic assemblages, sites yielding Doushantuo-type microscopic assemblages, which could help to resolve some of the most fundamental questions on the evolution of life and clarify the distribution of these organisms in the Ediacaran world, have proved elusive^14^.

Here we present the first record of Ediacaran Doushantuo-type microfossils from Laurentia (Portfjeld Formation, North Greenland). The Portfjeld biota consists of three-dimensionally preserved putative eggs and embryos, as well as acanthomorphic and leiosphaeric acritarchs, red algal thalli, sheet-like and oscillatoriacean cyanobacteria, and microbial mat fragments. The assemblage is directly comparable to similarly preserved fossils from the Doushantuo Formation but its significance at this time lies in greatly expanding the known record of Ediacaran phosphatized microfossils geographically, from the northern hemisphere of the Ediacaran world into the middle latitudes of its southern hemisphere^15,16^. In addition, the preservation of the Portfjeld biota in a shallow water setting greatly increases our insight into the environments where life evolved during the Ediacaran.

## Results and discussion

### Stratigraphic context of the Portfjeld Formation

**Fig. 1:**
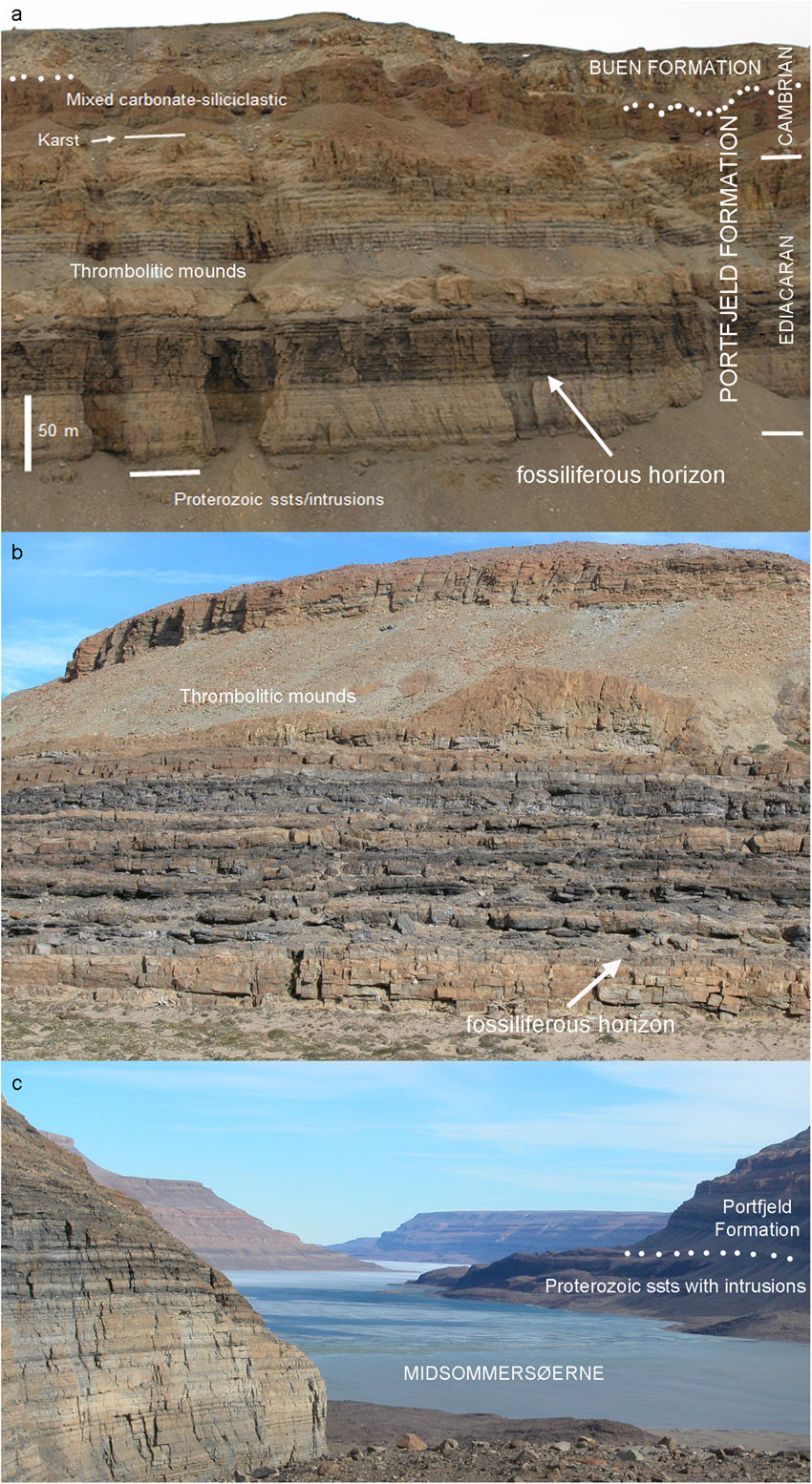
Field photographs of locality of the Portfjeld biota. **a** Portfjeld Formation—basal Buen sandstones at Midsommersøer, notice the conspicuous band of dark cherty dolomites. **b** Detail of Portfjeld Formation, west of Midsommersøer, with the same darky cherty dolomites overlain by thrombolitic mounds, showing the lithostratigraphic horizon yielding the Portfjeld biota. **c** View looking east along Wandel Dal with Midsommersøer, taken from the fossil locality (off shot left). Scale bar valid for **a**.

**Fig. 2:**
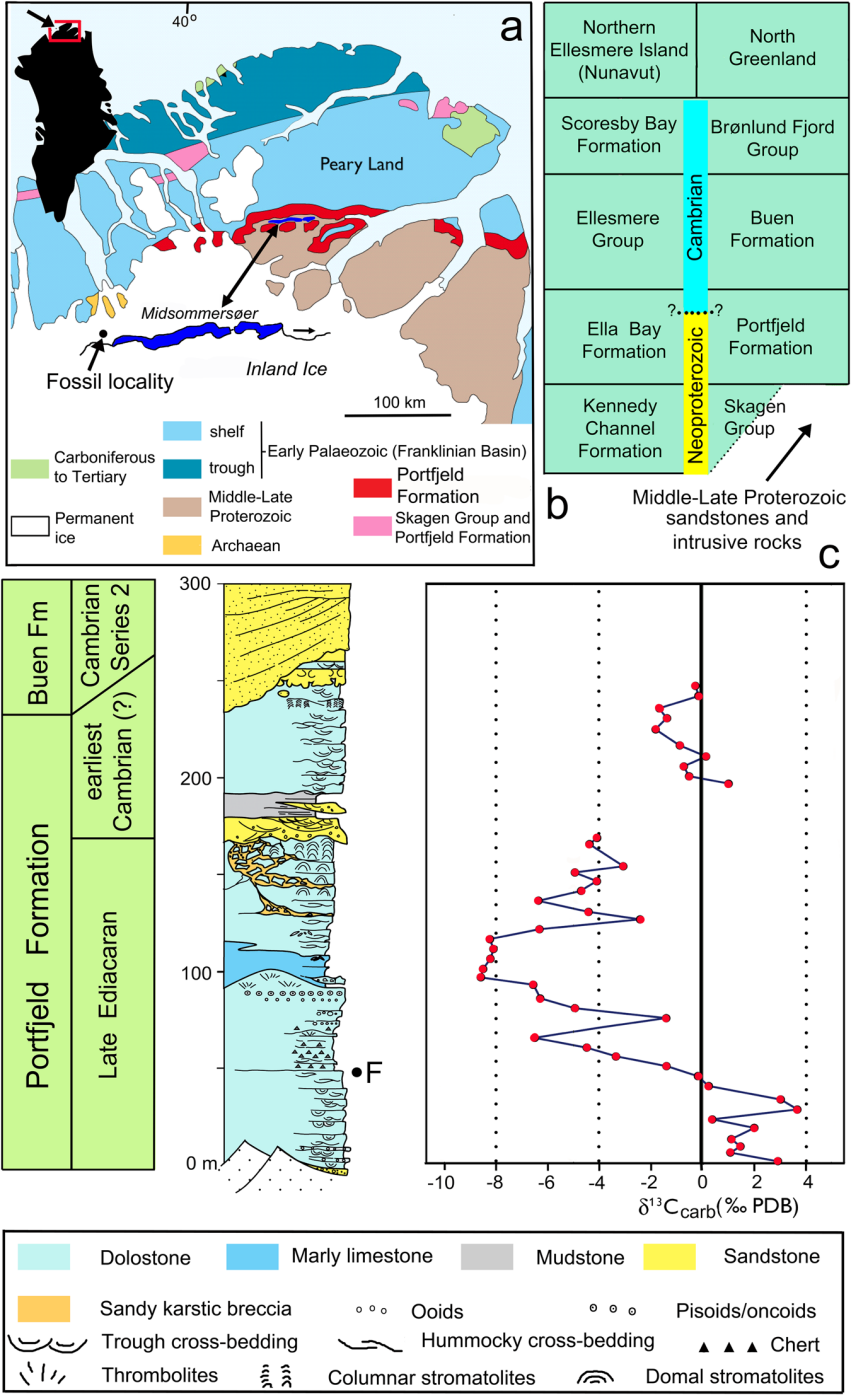
Geography, geology, and stratigraphy of the study area, North Greenland. **a** Geological map showing the sample locality at Midsommersøer, North Greenland. **b** Stratigraphic schemes in northern Ellesmere Island and North Greenland. **c** Stratigraphic section through the Portfjeld Formation at the western end of Midsommersøer compared with δ^13^C_carb_ (^o^/_oo_ PDB) values indicating the Shuram–Wonoka anomaly (about 570–560 Ma), “F” indicates the sample locality.

The Portfjeld Formation is the lowermost formation of the Franklinian Basin in southern Peary Land (Figs. 1 and 2a, b), resting unconformably on Mesoproterozoic sandstones of the Independence Fjord Group and localized erosionally truncated outliers of Neoproterozoic tillites and associated carbonates of inferred Marinoan affinity (for regional stratigraphic reviews, see^17,18^). The carbonate-dominated Portfjeld Formation is overlain, at a karstified unconformity (Fig. 2c), by transgressive fluvial to marine shelf siliciclastics of the Buen Formation. The sandstone-dominated lower member of the Buen Formation yields trace fossils of early Cambrian age^19^, while the mudstone-dominated upper member contains rich faunas of Cambrian Series 2 (Stage 3–4) age^20^.

The Portfjeld Formation comprises two discrete stratigraphic packages separated by a regionally developed karstic unconformity. The lower succession, about 170-m thick, is dominated by dolostones with rare limestones and represents two transgressive–regressive cycles of a carbonate ramp. Typical facies include hummocky cross-stratified intraclast-rich grainstones and cherty dark dolostones of the mid- and outer ramp, and ooid–pisoid grainstones and varied microbial facies of the inner ramp, including columnar and meter-scale domal stromatolites and thrombolitic bioherms. The capping hiatal surface shows penetrative and multi-generational karstic features extending some 40 m beneath the surface (Fig. 2c), including extensive interstratal solution, brecciation, and successive cave/vug/fracture fills and cementation, testifying to a protracted period of subaerial exposure. The transgressive succession of the upper Portfjeld Formation (*c*. 70–90 m thick) comprises fluvial sandstones and mudstones succeeded by high-energy shallow marine carbonate and siliciclastic facies, truncated upwards by dolines and karstic collapse structures at the Portfjeld–Buen formation boundary.

Chemostratigraphy shows that the δ^13^C of the Portfjeld Formation carbonate samples range from +4‰ to −8‰. Positive δ^13^C values persist over the lower c. 40 m of the formation, before a marked negative shift of 12‰ down to values of −8‰. A more gradual increase characterizes the δ^13^C values up-section through the karstified strata to the karstic unconformity at 167 m, following which there is a clear stabilization in δ^13^C values to values around 0 to −1‰ for the remainder of the succession (Fig. 2c).

The δ^13^C database for Neoproterozoic carbonate sections has proliferated within the last 30 years to the point where a δ^13^C compilation curve can act as a chemostratigraphic correlation tool for newly studied sections. Utilizing chemostratigraphy as a chronology tool involves the correlation of globally coherent geochemical perturbations and trends in vertical carbonate successions, within a broadly understood timeframe. This is particularly useful when attempting to refine age estimates for successions that lack abundant biostratigraphical and/or radiometric data. Utilizing the most up-to-date δ^13^C chemostratigraphic framework^21^, the asymmetric negative δ^13^C excursion and more gradual recovery displayed by the mid-section of the Portfjeld Formation can be aligned with the most extreme C-isotope variation recorded in Earth’s history: the Shuram–Wonoka anomaly. The form and magnitude (∼12‰) of this δ^13^C excursion, as well as a nadir value of −8‰, are unique to the Shuram–Wonoka anomaly and deter its alignment with other Neoproterozoic excursions, as well as the Basal Cambrian Isotope Excursion, BACE^21^. The Shuram–Wonoka anomaly is recognized intercontinentally in Late Ediacaran strata^22^ and provides a broad chronostratigraphic marker to constrain the biostratigraphy presented in this study. Williams and Schmidt^22^ noted that the Wonoka excursion spanned an interval of up to 10 Myr. from about 570 to 560 Ma and was recognized in shallow marine shelf environments on three palaeocontinents with low palaeolatitudes (<32°), whereas the North Greenland record reported here is from middle palaeolatitudes^15,16^.

### The Portfjeld biota

Well-preserved, phosphatized spiral oscillatoriacean cyanobacteria were recovered from strata in southern Peary Land and described by Peel^23^ (Fig. 2c, locality F). That study also noted the presence of smooth, wrinkled, or crumpled spheres resembling *Olivooides*, which prompted the present investigation. Consequently, we processed new fractions of the stromatolitic dolostone sample from the Portfjeld Formation. Here, we report some of the main findings within a diverse assemblage of microfossils that is comparable to the long-studied and highly important Doushantuo Formation biota of China.

Undisputed animal embryos were first identified from the Cambrian of China through the description of a series of developmental stages in *Olivooides* and *Markuelia*. Despite being simple in morphology there is good evidence from developmental series showing that at least some *Olivooides* develop into cnidarians^4,24,25^ but the simple spherical morphology of these earliest growth stages also permits other interpretations (e.g., echinoderms^8^). *Markuelia* is usually considered to be a scalidophoran^26^. Similarly, proposed animal cleavage embryos have been reported also from the older Ediacaran Doushantuo Formation, but their interpretation is contested with several hypotheses concerning their affinity still current^5,9,10,12^. The putative eggs and embryos described here are directly comparable in morphology and age to those from the Doushantuo Formation.

**Fig. 3:**
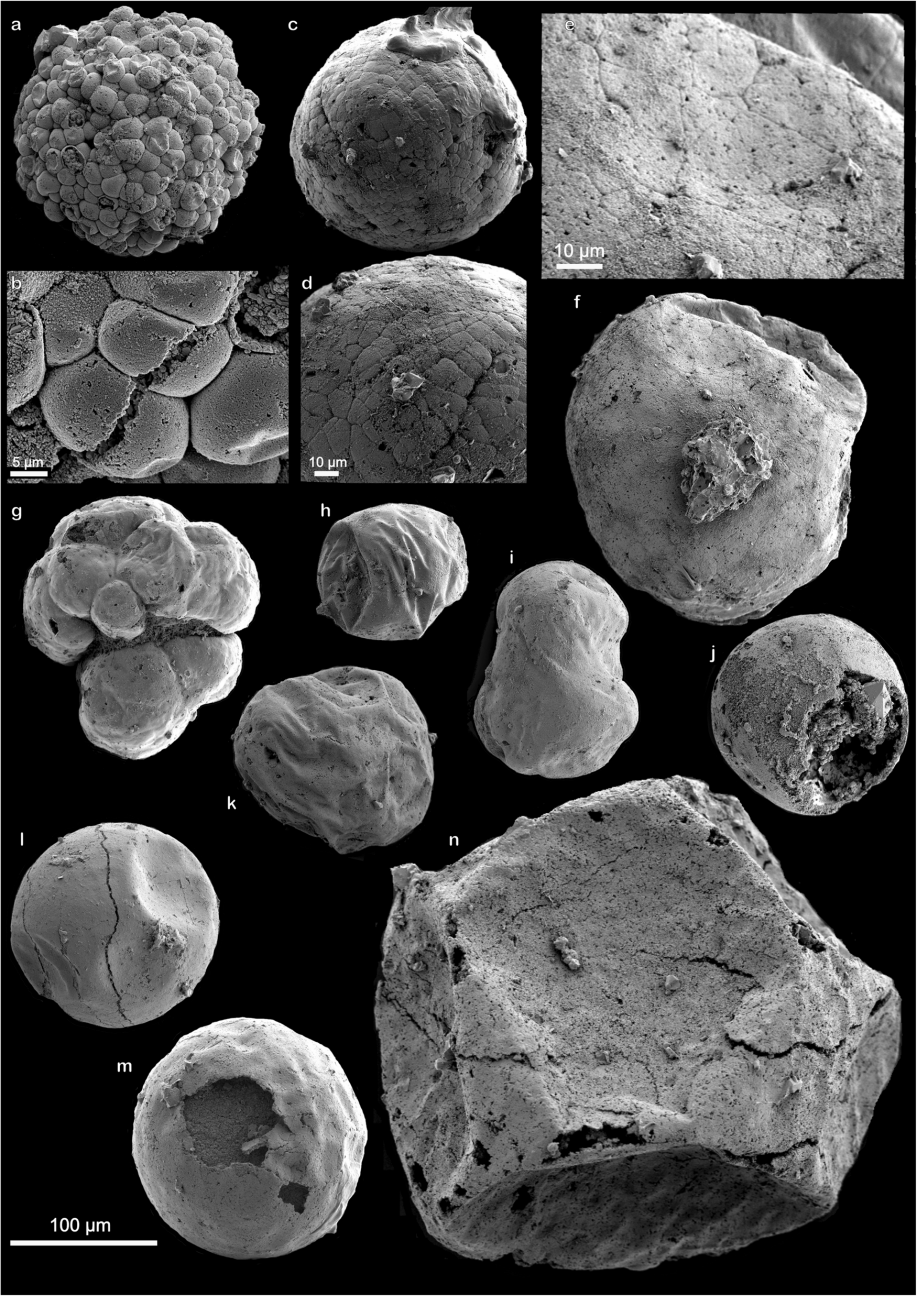
Putative eggs, embryos, and red algae from the Portfjeld Formation. **a–f** Putative cleavage embryos. **b, d, f** Enlarged to show the detail of polygonal cell junctions. **g** Red algal thalli similar to *Gremiphyca corymbiata*. **h–m** Putative eggs showing various degrees of taphonomic degradation (**h**, **k** ductile; **j**, **m** brittle). **i** Shows a possible peanut-shaped cell division. **j, m** Showing breakage of vesicle wall, the shape of the breakage, its size and location on the specimen is similar in many specimens and may therefore be interpreted as a biological feature rather than random breakage. **n** Shows a comparably large unidentified acritarch showing polygonal shrinkage and a golf ball-like vesicle surface structure. Scale bar 100 µm, unless where individually stated. Accession numbers; **a, b** PMU 36863/1. **c, d** PMU 36864/1. **e, f** PMU 36865/1. **f** PMU 36865/1. **g** PMU 36866/1. **h** PMU 36867/1. **i** PMU 36868/1. **j** PMU 36869/1. **k** PMU 36869/2. **l** PMU 36870/1. **m** PMU 36869/3. **n** PMU 36864/2.

**Fig. 4:**
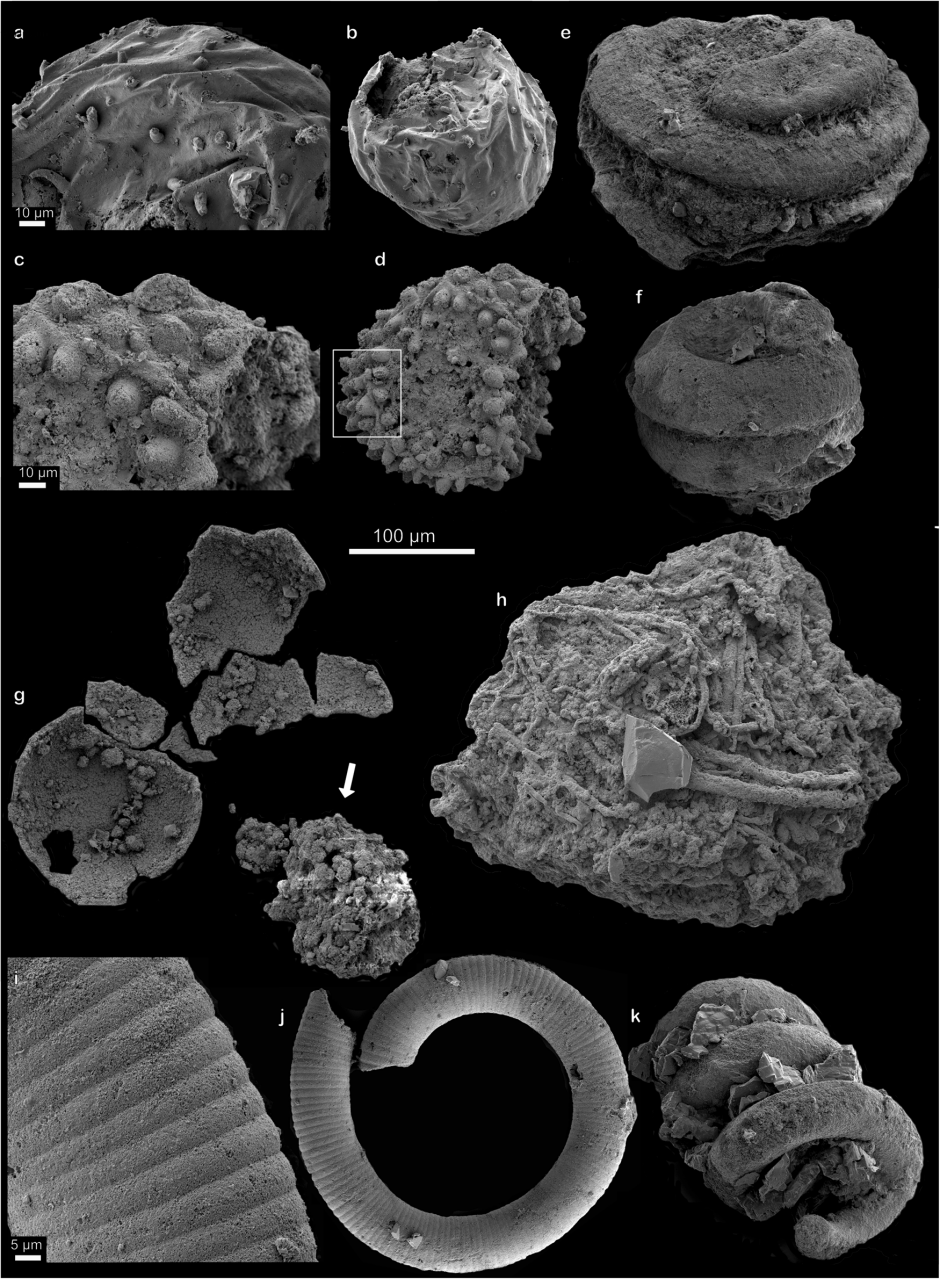
Acanthomorphic acritarchs, helically coiled spheroids, microbial mat fragment. **a**, **b** *Cavaspina acuminata*, showing nature of sparsely separated, tapering processes. **c**, **d** *Asterocapsoides wenganensis* showing densely arranged, conical processes. Box in **d** shows the conical shape of mostly unbroken processes. **e**, **f** Helically anti-clockwise coiling, spheroidal microfossils with closed termination. **g** Putative egg shell broken during preparation displaying internal contents (indicated by arrow). **h** Microbial mat displaying community structures with intertwined filamentous structures. **i**, **j** Coiled cyanobacterium *Jiangispirellus groenlandicus* and close up of individual cell walls. **k** *Spirellus shankari* cyanobacterium showing the nature of the coiling helix. Scale bar 100 µm, unless where individually stated. Accession numbers; **a, b** PMU 36871/1. **c**, **d** PMU 36872/1. **e** PMU 36866/2. **f** PMU 36866/3. **g** PMU 36872/2. **h** PMU 36873/1. **i**, **j** PMU 36873/2. **k** PMU 36874/1.

**Fig. 5:**
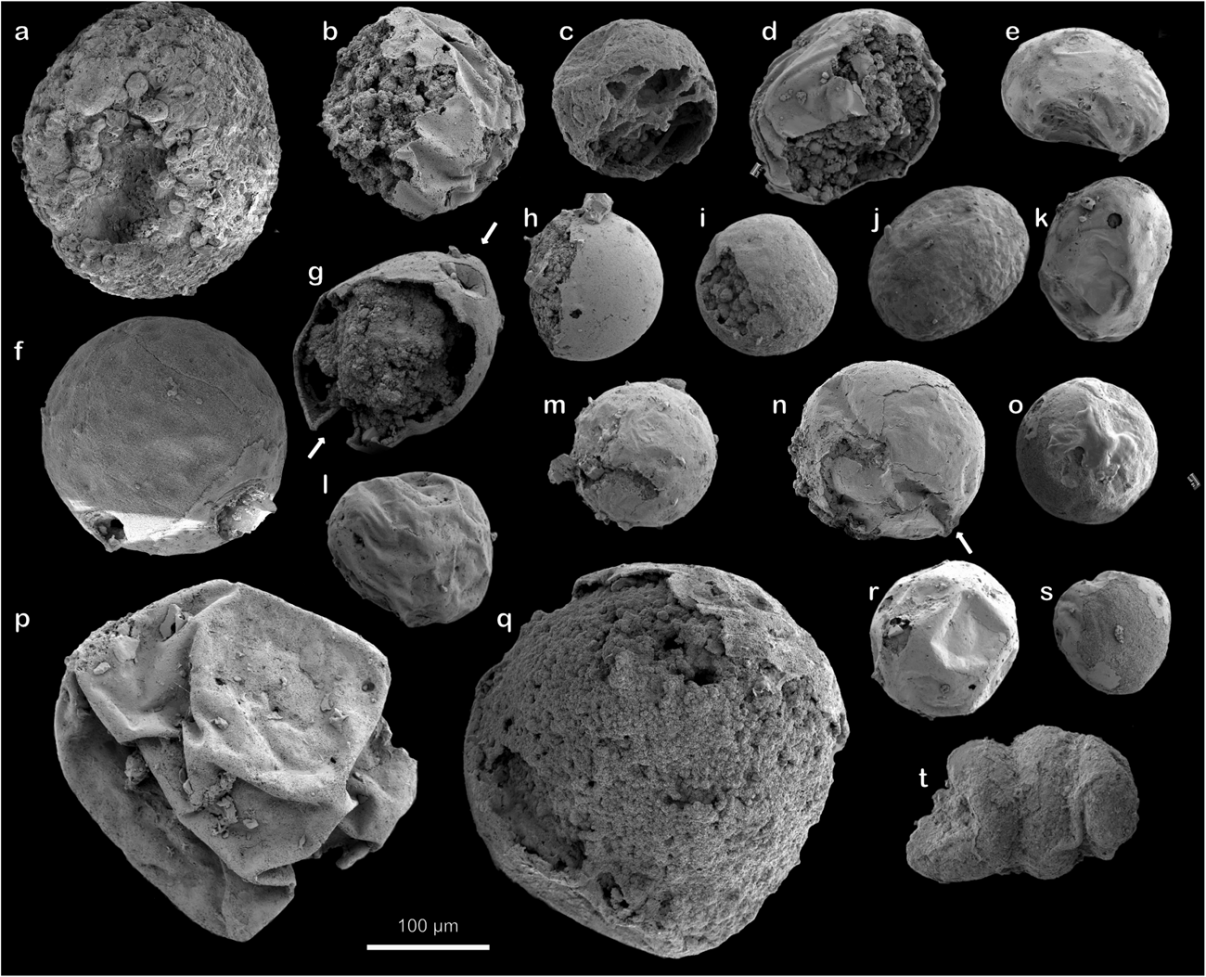
Putative eggs and embryos at various stages of taphonomic degradation. **a** Putative cleavage embryo displaying individual cells. **b–s** Putative eggs/embryos in various states of degradation; internal contents are often preserved and seen as external enveloping layer is broken or peeled off (**b–d, g–i, q**) but absent when compressed (**p**); surface structures vary from golf ball-like (**f**), wrinkled (**b**), pitted (**j**) to smooth (**h**), and from thin-, (**s**) to thick-walled (**g**). Some specimens have surface structures that may represent grooves (arrow in **n**) or polar invaginations (arrows in **g**). **t** Probable tightly coiled cyanobacterium (but see also Fig. 6 in Yin et al.^13^ for similar pseudo-uncoiling in *Helicoforamina*). Scale bar 100 µm. Accession numbers; **a** PMU 36868/2. **b** PMU 36869/4. **c** PMU 36874/2. **d** PMU 36875/1. **e** PMU 36876/1. **f** PMU 36876/2. **g** PMU 36877/1. **h** PMU 36875/2. **i** PMU 36877/2. **j** PMU 36877/3. **k** PMU 36874/3. **l** PMU 36869/5. **m** PMU 36876/3. **n** PMU 36872/2. **o** PMU 36878/1. **p** PMU 36864/3. **q** PMU 36865/2. **r** PMU 36879/1. **s** PMU 36877/4. **t** PMU 36868/2.

Biologically, the transition from egg to embryo comes at fertilization, after which the egg enters the reproductive stage. In fossil material this distinction is normally seen as a ball of cells, where the number of cells doubles during each division. Spheroidal microfossils with smooth envelopes recovered from Portfjeld Formation are interpreted as putative eggs. The embryo-like fossils from the Portfjeld biota consists of clusters (150–170 µm in diameter) of hundreds of individual cells, normally 15–20 µm in diameter, but many are smaller (5–10 µm in diameter). The cells are tightly packed and seem to extend inwards. Neighboring cells appear to accommodate each other, indicating that they are not rigid algal clusters. Most cells are complete but show evidence of deflation or, where broken, display internal phosphatized contents. One specimen (Fig. 3a, b) is interpreted as late stage “*Megaclonophycus*-stage” (compare Fig. 2G in^27^). Two others (Fig. 3c–f) are similar to cleavage embryos (256-cell or similar) reported from the Cambrian Kuanchuanpu Formation in China (compare Fig. 5 in^28^) but comparisons can also be made with *Wengania globosa* and *Wengania exquisita* from the Ediacaran Weng’an biota^29^. A morphological furrow may be present. A peanut-shaped specimen (Fig. 3i) can be compared with the germinating stage in *Tianzhushania*^10^, although this simple morphology is not sufficient in itself to make such a definite link. Taxonomic details have been examined in various contexts with regards to suites of developmental stages (for example referring all developmental stages including *Megasphaera*, *Parapandorina*, *Megaclonophycus*, and *Yintianzhushania* to *Tianzhushania*) and we refrain from commenting further on this here, but see^12,30–32^ for discussion. Many vesicles are hollow and show evidence of flexible deformation during deflation prior to phosphatization (Fig. 3h, k). Others, which are more delicate, break during mounting to reveal the originally organic internal contents (Fig. 4g). Many other smooth vesicles show evidence for pre-determined rupture (Fig. 3j, m). Simple, lobose, pseudoparenchymatous thalli (Fig. 3g) resembling florideophyte red algae (*Gremiphyca corymbiata*)^29^ are also present in the Portfjeld biota.

In addition to fossils previously interpreted as eggs and embryos, acanthomorphic acritarchs are rare but well-preserved and important constituents of the Portfjeld biota. *Cavaspina acuminata* (Fig. 4a, b) is a spheroidal vesicle ~160 µm in diameter with solid and widely separated processes tapering to a conical tip that seems to curve. About 40 processes, many of which are broken, are visible on the vesicle surface (unbroken processes ~15 µm long, which is 10% of vesicle diameter, width at base 5 µm). The originally spheroidal (long axis ~185 µm) *Asterocapsoides wenganensis* (Fig. 4c, d) is characterized by its short, hollow, homomorphic, evenly distributed, conical processes that taper to a sub-rounded tip (processes are 15–20 µm long, 10–15 µm at base and 5 µm at the tip, and spaced 5 µm apart). Similar acanthomorphic acritarchs (e.g., *Mengeosphaera* with biform processes, or *Meghystrichosphaeridium*, with pentagonal or hexagonal fields around the processes) are described from Doushantuo, displaying a taphonomic and taxonomic connection between the Portfjeld and the Doushantuo biota. Spheroidal vesicles, slightly compressed at the poles (diameter 160–180 and 210–300 µm, individual whorls ~50–60 µm in thickness), consisting of three, anti-clockwise coiling whorls, with a rounded termination are rare but well-preserved, and indicative of early biological chirality (Fig. 4e, f). Similar fossils, although larger and with clockwise coiling, were described as the later-stage part of a developmental series (compare Fig. 1O in^33^)^13^.

Helically coiled filamentous microfossils are very common remains in the Portfjeld biota; they are also well represented in the Khesen Group of Mongolia^14^, but rare in the Doushantuo Formation. Three main types, *Obruchevella*, *Spirellus*, and *Jiangispirellus*, all interpreted as oscillatoriacean cyanobacteria, were initially described^23^. *Jiangispirellus groenlandicus* (Fig. 4i, j) consists of an open-coiled trichome with delicately preserved cell structure. *Spirellus shankari* (Fig. 4k) consists of a helix without evidence of cell structure and is interpreted as a filament that is often calcified (now phosphatized). The different types of cyanobacteria show a range in differential taphonomic preservation representing degrees of degradation and mineralization of the original form.

### Evolutionary importance of the Portfjeld biota

Peel^23^ considered the oscillatoriacean cyanobacteria within the Portfjeld biota to be consistent with an early Cambrian age following geological correlation with the Ella Bay Formation of easternmost Ellesmere Island (Nunavut, Canada; Fig. 2b), where samples with early Cambrian macrofossils were known at a lower stratigraphic level^34^. However, the fossils reported by Long^34^ from below the Ella Bay Formation, the direct lithological correlative of the Portfjeld Formation along the northern coast of Greenland, were demonstrated subsequently to have been tectonically emplaced from overlying strata of the Cambrian Ellesmere Group^35^ (Fig. 2b). In consequence, prior to the present discoveries, biostratigraphic control of the age of the Ella Bay and Portfjeld formations was restricted to early Cambrian fossils and trace fossils occurring above the formations in Ellesmere Island and North Greenland^18,20,35^. A late Neoproterozoic age for the Portfjeld and Ella Bay formations was proposed tentatively by Dewing et al.^35^ on the basis of correlation with the Risky Formation of the Mackenzie Mountains, northwestern Canada, and the Spiral Creek Formation of North-East Greenland. The age of the former is constrained by Ediacaran macrofossils^36^, whereas the Spiral Creek Formation is a correlative of successions in eastern Svalbard yielding Neoproterozoic acritarchs^37^.

The δ^13^C values in the uppermost carbonates of the Portfjeld Formation, above the karstic unconformity, are compatible with global early Cambrian values. This confirms the interpretation that the intra-Portfjeld unconformity represents a substantial depositional hiatus, supporting the view that the Portfjeld Formation spans the Precambrian–Cambrian boundary^18^.

Simple multicellular organisms may have evolved already in the Mesoproterozoic^38^ but it was first in the Ediacaran that complex eukaryotes began to diversify. Until now, the Doushantuo Formation has been our main source of information on soft-bodied organisms predating the classical and enigmatic, macroscopic Ediacaran biota^12,39^. As such, the discovery of the same type of fossils from North Greenland offers important additional evidence to understanding soft-bodied organismal evolution. The many spheroidal fossils (Fig. 5) discovered in the Portfjeld biota show a variety of morphologies consistent with interpretations that conform well with both blastula stage embryos and spiral stage embryos^13^. The exact phylogenetic framework is complex; multicellularity, for example, evolved on many different occasions and independently in animals, fungi, and algae^40,41^. Modern animal embryos or volvocine green algae may provide analogs to Ediacaran embryos but these interpretations are nevertheless imperfect; it is unlikely that the fossils described here represent crown-group animals. The Portfjeld acritarchs form part of a globally distributed and diverse assemblage of morphologically complex and well-documented acritarchs described from carbonaceous compressions in shales, from thin-sectioned cherts as well as phosphatized^42–48^. As with the eggs and embryos, phylogenetic uncertainties must be resolved through study of available material from all assemblages.

The Portfjeld biota seems to preserve specimens that are generally smaller (~100–200 µm) than their morphologically similar Doushantuo biota counterparts (~400–500 µm or larger). There may be several reasons for this discrepancy but they all fall within the framework of natural variation. For the embryo-like fossils, ontogeny may play a role but more importantly the geographic distance between Portfjeld and Doushantuo and the differences in their environments of accumulation must also be taken into consideration.

**Fig. 6:**
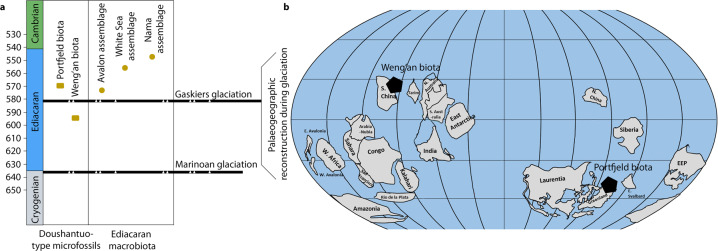
Stratigraphic and palaeogeographic distribution of evolutionary important Ediacaran assemblages. **a** Stratigraphic distribution of the Portfjeld biota and the Weng’an biota (representative of Doushantuo Formation), the latter of which predates the classical macroscopic “Ediacaran biota” and probably also predating the Gaskiers glaciation^39^. The potential stratigraphic range of the biotas is indicated by the vertical line but detailed stratigraphic correlation and relationships to other biotas remain to be resolved. **b** Palaeogeographic reconstruction at ca. 580 Ma, black polygons show location of the two taphonomic windows of this type known from the Ediacaran and their spatial separation (adapted from^49,54^).

Palaeogeographically, South China (including the Yangtze Block and the Doushantuo Formation) was probably drifting southwards from a low northerly latitude at the time of deposition of the Weng’an biota, usually suggested to be ~580 Ma ago^49,50^, although it could be as old as 609 ± 5 Ma^51^. Laurentia (including North Greenland and the Portfjeld Formation) is estimated to have lain at palaeolatitudes of 30–75° S, with Laurentia completely isolated from all other continents (Fig. 6). Thus, Laurentia and South China were significantly separated from each other at the time of deposition of the two biotas, lying in different hemispheres. While the Weng’an biota yields the oldest putative metazoans, the discovery of similar fossils from the Portfjeld Formation, half a world away, demonstrates that these early possible animals had a worldwide distribution. The palaeogeographic separation is evident but not surprising given the global distribution of many other important Ediacaran fossils. It is therefore perhaps a question of propitious preservation rather than geographic constraints. The two biotas are seemingly older than most of the classic, enigmatic macrofossil biotas now known globally (Fig. 6), with the potential exception of the Avalon biota (574–564 Ma^52^). Furthermore, they occupied different environments with the Portfjeld biota deposited in an inner carbonate shelf environment^18^, whereas the Weng’an biota accumulated on the outer shelf^39,53^. The driving force behind this early evolution has often been attributed to ocean oxidation and fluctuations in the marine carbon and sulfur cycles, but the successful establishment of these early ecosystems may have been dependent on local environmental fluctuations^54^. However, the discovery of the Portfjeld biota indicates that this early evolution was not restricted locally to China, nor to outer shelf environments, but flourished in geographically separated areas, as was the case also with the younger macrobiota as well as the acanthomorphic acritarchs ranging throughout the Ediacaran. Thus, oxygenation was probably widespread at this time, providing new direct evidence about the early evolution of cellularly differentiated eukaryotes and even the early evolution of animals.

It is evident that the Portfjeld biota predates the nadir of the Shuram–Wonoka anomaly (Fig. 2c). However, there is no geochemical or lithostratigraphic evidence in the measured Portfjeld section to suggest that this succession encompasses the Gaskiers Glaciation (580 Ma)^55^, indicating that Portfjeld biota, age wise, lies between the Shuram–Wonoka anomaly and the Gaskiers Glaciation (Fig. 5a) and likely preserves a different evolutionary scenario. Published age estimates for the Weng’an biota range from ~580 Ma ago^49,50^ to 609 ± 5 Ma^51^ and suggest that the Weng’an biota may be at least 10 Ma or possibly as much as 40 Ma older than the Portfjeld biota. Given the magnitude of the age difference, however uncertain, the degree of palaeogeographic separation of the localities and their contrasting environments, it is evident that the Portfjeld biota provides an additional window onto the early evolution of Ediacaran life, rather than a mere duplication of the Weng’an event.

## Conclusions

The Portfjeld Formation crops out over hundreds of kilometers in North Greenland but is poorly known on account of its remoteness. The assemblage of extremely well-preserved microfossils presented here, and its striking similarity to previously described fossils from the Doushantuo Formation of China, demonstrates greater complexity and worldwide distribution of the late Ediacaran ecosystem than previously recognized. The finds from North Greenland extend the known distribution of the Ediacaran Doushantuo-like biota along the length of the Pannotian palaeocontinent, from low to middle latitudes in the northern hemisphere (China) to the middle latitude position in the southern hemisphere occupied by North Greenland in eastern Laurentia; their age is confirmed by chemostratigraphy.

With a background in the largely unexplored potential represented by the Portfjeld Formation, the new discoveries offer excellent prospects for resolving the phylogenetic relationships of many of these problematic multicellular Ediacaran eukaryotes and a better understanding of the environments in which they evolved.

## Methods

### Material

The fossiliferous sample was collected by John S. Peel and Peter Frykman in 1978 from the lower succession, about 50 m above the base of the formation (equivalent to c. 50 m on Fig. 1c) on the north side of Wandel Dal, west of Øvre Midsommersø (82°14’ N, 36°06’ W, Fig. 1). Samples for stable isotope geochemistry were collected in 2006 at c. 5 m intervals throughout the carbonate intervals of the formation. All collection activities occurred within the Danish State and no international transport or permits were required.

Stromatolitic dolostone rock fragments from sample 271770, a split of the original sample 271769 processed by Peel^23^, were macerated in weak acetic or formic acid and sieved in fractions down to 44 µm. The residues containing phosphatized microfossils were hand-picked under a binocular microscope. Specimens were mounted on aluminium stubs and imaged under high power using a Zeiss Supra 35VP scanning electron microscope. Images were cropped using Affinity Photo and Affinity Design.

### Geochemistry/isotopes

Carbon isotopes were analyzed at the Department of Geography and Geology, University of Copenhagen, using a Micromass mass spectrometer equipped with a multiflow gas bench inlet preparation system. CO_2_ was liberated by reaction with purified phosphoric acid at 80 °C. Calibrations to V-PDB standard via NBS-19 were performed using the Copenhagen in-house marble standard (LEO). Reproducibility of replicated standards is better than 0.1‰ for δ^13^C. Data are reported using the conventional δ notation to indicate the ‰ deviation from the arbitrary V-PDB (Vienna-Pee Dee Belemnite) standard.

### Reporting summary

Further information on research design is available in the Nature Research Reporting Summary linked to this article.

## Supplementary information

Reporting Summary

## Data Availability

All imaged fossil material is deposited at the Paleontological collections of Museum of Evolution (PMU) Uppsala University, Sweden. Accession numbers are provided for each fossil using a PMU number. Geochemical datasets are available upon request by contacting Robert Frei (robertf@ign.ku.dk).
