# Supplementary material for: Ediacaran Doushantuo-type biota discovered in Laurentia
Source: Commun Biol. 2020 Nov 6;3:647. doi: 10.1038/s42003-020-01381-7 (PMC7648037; doi:10.1038/s42003-020-01381-7)
Supplement: Supplementary file 1 — Reporting Summary [file 42003_2020_1381_MOESM1_ESM.pdf]

nature research

Corresponding author(s): Sebastian Willman

Last updated by author(s): Oct 4, 2020

Reporting Summary

Nature Research wishes to improve the reproducibility of the work that we publish. This form provides structure for consistency and transparency in reporting. For further information on Nature Research policies, see our Editorial Policies and the Editorial Policy Checklist.

Statistics

For all statistical analyses, confirm that the following items are present in the figure legend, table legend, main text, or Methods section.

n/a	Confirmed	
☒	☐	The exact sample size (n) for each experimental group/condition, given as a discrete number and unit of measurement
☒	☐	A statement on whether measurements were taken from distinct samples or whether the same sample was measured repeatedly
☒	☐	The statistical test(s) used AND whether they are one- or two-sided<br>*Only common tests should be described solely by name; describe more complex techniques in the Methods section.*
☒	☐	A description of all covariates tested
☒	☐	A description of any assumptions or corrections, such as tests of normality and adjustment for multiple comparisons
☒	☐	A full description of the statistical parameters including central tendency (e.g. means) or other basic estimates (e.g. regression coefficient) AND variation (e.g. standard deviation) or associated estimates of uncertainty (e.g. confidence intervals)
☒	☐	For null hypothesis testing, the test statistic (e.g. F, t, r) with confidence intervals, effect sizes, degrees of freedom and P value noted<br>*Give P values as exact values whenever suitable.*
☒	☐	For Bayesian analysis, information on the choice of priors and Markov chain Monte Carlo settings
☒	☐	For hierarchical and complex designs, identification of the appropriate level for tests and full reporting of outcomes
☒	☐	Estimates of effect sizes (e.g. Cohen's d, Pearson's r), indicating how they were calculated

Our web collection on statistics for biologists contains articles on many of the points above.

Software and code

Policy information about availability of computer code

Data collection	Scanning electron microscope, Photoshop CS
Data analysis	No software used

For manuscripts utilizing custom algorithms or software that are central to the research but not yet described in published literature, software must be made available to editors and reviewers. We strongly encourage code deposition in a community repository (e.g. GitHub). See the Nature Research guidelines for submitting code & software for further information.

Data

Policy information about availability of data

All manuscripts must include a data availability statement. This statement should provide the following information, where applicable:

- Accession codes, unique identifiers, or web links for publicly available datasets
- A list of figures that have associated raw data
- A description of any restrictions on data availability

Fossil repository at the Museum of evolution in Uppsala. Fossil will be provided with museum numbers before publishing.

April 2020

Field-specific reporting

Please select the one below that is the best fit for your research. If you are not sure, read the appropriate sections before making your selection.

☐ Life sciences ☐ Behavioural & social sciences ☒ Ecological, evolutionary & environmental sciences

For a reference copy of the document with all sections, see nature.com/documents/nr-reporting-summary-flat.pdf

Ecological, evolutionary & environmental sciences study design

All studies must disclose on these points even when the disclosure is negative.

Study description	A description of fossil material containing putative embryos from the Ediacaran Portfjeld Formation, North Greenland.
Research sample	Stromatolitic dolostone rock fragments were macerated in weak acetic or formic acid. The sample was chosen because it had previously been shown to contain the fossil Olivooides.
Sampling strategy	Samples were collected during 1978 by the Geological Survey of Greenland during its routine geological investigations and mapping activities in Greenland.
Data collection	Fossils were hand-picked from residue and imaged using a scanning electron microscope.
Timing and spatial scale	N/A
Data exclusions	N/A
Reproducibility	Details about locality and stratigraphic position is noted to ensure reproducibility.
Randomization	N/A
Blinding	N/A

Did the study involve field work? ☒ Yes ☐ No

Field work, collection and transport

Field conditions	Geological rock samples collected in late July 1978 (short summer field season expedition) from cliff exposures. Operative conditions included 24 hour day light and temperatures in the 5-10 degrees C range. No precipitation.
Location	82 degrees 14'N, 36 degrees 06'W, at a height of about 400 m above sea level on a south-facing slope, in a high arctic true desert environment characterised by bare rock and talus surfaces; no vegetation cover.
Access & import/export	Samples were collected in accordance with established procedures ("Field instructions") of the Geological Survey of Greenland and transported to Copenhagen together with other expedition materials and personnel following the demobilization of the expedition in August 1978. All activities occurred within the Danish State and no international transport or permits were required. Collection and transport of samples lay within the responsibilities of the Geological Survey of Greenland as the Danish Government directorate (Ministry of Greenland) officially authorized to conduct geological investigations and mapping of Greenland.
Disturbance	No visible effect on collecting site

Reporting for specific materials, systems and methods

We require information from authors about some types of materials, experimental systems and methods used in many studies. Here, indicate whether each material, system or method listed is relevant to your study. If you are not sure if a list item applies to your research, read the appropriate section before selecting a response.

Materials & experimental systems

n/a	Involved in the study
☒	☐ Antibodies
☒	☐ Eukaryotic cell lines
☐	☒ Palaeontology and archaeology
☒	☐ Animals and other organisms
☒	☐ Human research participants
☒	☐ Clinical data
☒	☐ Dual use research of concern

Methods

n/a	Involved in the study
☒	☐ ChIP-seq
☒	☐ Flow cytometry
☒	☐ MRI-based neuroimaging

Palaeontology and Archaeology

Specimen provenance

The rock sample was collected by survey personnel in southern Peary Land, North Greenland, during routine regional geological investigations of the Geological Survey of Greenland (Grønlands Geologiske Undersøgelse; GGU) as part of its official "North Greenland Project 1978-80". Rock samples, including the present samples, and fossils were routinely collected to characterize the geological units present within the project area. At that time, the Geological Survey of Greenland was a Directorate within the Ministry of Greenland of the Danish Government, which was charged with the systematic geological mapping and all geological investigations of Greenland. All activities were in accordance with its rights and responsibilities in accordance with this function. In recent years, the Geological Survey of Greenland was incorporated into the Geological Survey of Denmark and Greenland, also within the Danish Government.

Specimen deposition

The specimens will be housed in the collections of the Museum of Evolution in Uppsala, Sweden

Dating methods

An Ediacaran age is determined through lithostratigraphic and carbon istotopic correlation.

☐ Tick this box to confirm that the raw and calibrated dates are available in the paper or in Supplementary Information.

Ethics oversight

No ethical approval has been required for either the collection or study of the present material

Note that full information on the approval of the study protocol must also be provided in the manuscript.
